# Cleansing the Teeth

**Published:** 1854-04

**Authors:** T. D. Thompson

**Affiliations:** Providence, R. I.


### ARTICLE IX.

*Cleansing the Teeth.* By T. D. THOMPSON, Providence, R. I.

THE different methods practiced by dentists, or persons calling themselves such, for removing the tartar from the teeth, or cleansing them, should, we think, be noticed. The good or evil resulting from these operations may be direct or remote.

The immediate or direct beneficial effects exerted upon the teeth, gums and sympathising parts, are such, usually, as to repay fourfold all labor thus expended; the teeth are freed from an injurious and corroding agent; the gums assume again their healthy action, and the air, the purity of which is so essential to a healthy circulation of the blood, is inhaled without contamination; in fact patients, and all who may be so situated as to receive the odor of the breath, at once perceive the grateful change. These, we conceive, to be some of the legitimate results arrising from a thorough and efficient cleansing of the teeth.

There are evils, likewise, to be noticed resulting from these operations. These injurious consequences do not result from the faithful and *honest* performance of these operations; but from a practice at once *dishonest*, and one which, we think, calls for the most decided reproof.

We believe where such is the known practice, the civil law should be invoked to punish the offender and *prevent* the infliction of injury that cannot be repaired.

Acid is often used as an agent in cleansing the teeth, or to remove the tartar from these organs. This is a common practice with some individuals; and instances are not uncommon where sets of valuable teeth have thus been mutilated, and even destroyed.

We have noticed the result of this practice on the incisor and cuspid teeth more particularly. We have frequently seen these teeth so much wasted as to be beyond the reach of remedial treatment.

We saw a young female, fifteen years of age, whose incisor and cuspid teeth had been destroyed, by the application of acid to cleanse them, in one year's time. This *valuable* operation was performed by a *dentist*, who advertised to cleanse the teeth "without scraping." This is by no means an isolated case; we have witnessed similar results from the use of diluted mineral acid, in cases of individuals in more advanced life.

We have referred to the remote effects of the improper treatment of the teeth—one case which has recently come to our notice, will be sufficient to illustrate what we mean.

A lady visited our office to have removed several parts of teeth, in either jaw, (her mouth was nearly destitute of teeth.) After complying with her request, she remarked that she attributed the destruction of her teeth to a dentist who made use of acid in cleansing them. The teeth had crumbled away, and the sharp angles had irritated her mouth very much. She showed me her tongue, on the left side of which, very near the apex, was a morbid growth about the size of a chestnut, its color and general appearance was that of a cancer. She said the formation commenced soon after her teeth began to decay, as though it arose from the irritation of her tongue against the sharp angles of the decayed teeth. Her views of the case appeared to us to be correct; what the termination may be, time will decide.

What we wished to express by the remote effects of mal-practice in cleansing the teeth, is we believe fully illustrated by the last cited case. This person was suffering not from the loss of her teeth alone, but in addition, she was tormented with this morbid growth upon the tongue, which may eventually cause the destruction of her life.

If in the preceding remarks we have drawn a fair inference, what can be said of that individual who will thus tamper with the human system, who will thus mutilate such valuable organs as the teeth.

